# Maternal nutrition modifies trophoblast giant cell phenotype and fetal growth in mice

**DOI:** 10.1530/REP-14-0667

**Published:** 2015-06

**Authors:** Adam J Watkins, Emma S Lucas, Stephanie Marfy-Smith, Nicola Bates, Susan J Kimber, Tom P Fleming

**Affiliations:** 1 Centre for Biological Sciences, Southampton General Hospital, University of Southampton, Southampton, SO16 6YD, UK; 2 Ageing School of Life and Health Sciences, Aston Research Centre for Healthy, Aston University, Birmingham, B4 7ET, UK; 3 Faculty of Life Sciences, University of Manchester, Michael Smith Building, Oxford Road, Manchester, M13 9PT, UK

## Abstract

Mammalian placentation is dependent upon the action of trophoblast cells at the time of implantation. Appropriate fetal growth, regulated by maternal nutrition and nutrient transport across the placenta, is a critical factor for adult offspring long-term health. We have demonstrated that a mouse maternal low-protein diet (LPD) fed exclusively during preimplantation development (Emb-LPD) increases offspring growth but programmes adult cardiovascular and metabolic disease. In this study, we investigate the impact of maternal nutrition on post-implantation trophoblast phenotype and fetal growth. Ectoplacental cone explants were isolated at day 8 of gestation from female mice fed either normal protein diet (NPD: 18% casein), LPD (9% casein) or Emb-LPD and cultured *in vitro*. We observed enhanced spreading and cell division within proliferative and secondary trophoblast giant cells (TGCs) emerging from explants isolated from LPD-fed females when compared with NPD and Emb-LPD explants after 24 and 48 h. Moreover, both LPD and Emb-LPD explants showed substantial expansion of TGC area during 24–48 h, not observed in NPD. No difference in invasive capacity was observed between treatments using Matrigel transwell migration assays. At day 17 of gestation, LPD- and Emb-LPD-fed conceptuses displayed smaller placentas and larger fetuses respectively, resulting in increased fetal:placental ratios in both groups compared with NPD conceptuses. Analysis of placental and yolk sac nutrient signalling within the mammalian target of rapamycin complex 1 pathway revealed similar levels of total and phosphorylated downstream targets across groups. These data demonstrate that early post-implantation embryos modify trophoblast phenotype to regulate fetal growth under conditions of poor maternal nutrition.

## Introduction

The development of the mammalian fetus is fundamentally dependent upon successful implantation of the embryo into the uterine tissue. This essential step is regulated by the action of the trophoblast cells that coordinate the complex processes of embryo attachment and implantation, and uterine spiral artery remodelling, and initiate the development of the placenta. At the time of implantation, the embryo comprises two distinct cell lineages, the inner cell mass (ICM) containing the progenitor cells of the fetal and primitive endoderm lineages, and the trophectoderm (TE) comprising the progenitors of the chorioallantoic placenta. Following implantation, TE cells not in direct contact with the ICM (mural TE) exit mitosis, endo-reduplicate their DNA and give rise to the primary trophoblast giant cells (TGCs). These primary TGCs form blood sinuses at the periphery of the embryo, contributing to the provision of nutrients and oxygen to the developing embryo by the yolk sac (Bevilacqua & Abrahamsohn 1988). In contrast, the TE cells directly overlying the ICM (polar TE) differentiate into the chorion and ectoplacental cone (EPC), from which secondary TGCs are derived (Simmons & Cross 2005).

The large polyploid secondary TGCs invade into the maternal decidual tissue and spiral arteries to establish maternal–fetal nutrient transfer (Adamson *et al*. 2002) as well as secreting a range of hormones and cytokines that modulate the maternal uterine environment, both essential for fetal growth (Simmons *et al*. 2007, Soares *et al*. 2007). At around day 8.5 of gestation in the mouse (E8.5), the allantois begins to attach to the chorion initiating the formation of both the junctional and labyrinth zones of the developing placenta. Ultimately, the labyrinth zone will mediate nutrient exchange between the fetus and mother through a highly developed vascular network, while the junctional zone becomes the site of growth factor and pregnancy-related hormone production (Georgiades *et al*. 2002). Therefore, disruption in the normal process of trophoblast differentiation or phenotype specification during early gestation (E8.5) is likely to affect detrimentally embryo viability, placental morphology and function and ultimately fetal growth and development.

Over recent decades, the regulation of fetal growth and development has gained significant interest in light of human epidemiological and animal model observations, demonstrating links between altered fetal growth and predisposition to adult-onset cardiovascular and metabolic diseases (Hanson & Gluckman 2014). The significant similarities between human and rodent preimplantation embryo development, implantation and placental morphology mean that mice and rats provide biologically applicable models with which to investigate the sensitivity of the developing placenta and its impact on offspring health. In mice, maternal caloric restriction results in differential placental expression of genes associated with intra-uterine growth restriction and genome-wide hypomethylation (Chen *et al*. 2013), while a high-fat diet induces sex-specific changes in placental gene expression for immune and inflammatory responses and regulators of epigenetic modifications (Gabory *et al*. 2012). Changes in placental gene expression within apoptosis, growth inhibition and epigenetic modification pathways have also been observed in response to a maternal low-protein diet (LPD) in mice (Gheorghe *et al*. 2009), while, in the rat, changes in placental mammalian target of rapamycin complex 1 (mTORC1) and amino acid transporter protein levels have been demonstrated (Rosario *et al*. 2011). Additional changes in placental structure, nutrient transport and fetal cytokine levels have also been reported in response to maternal dietary manipulation (Armitage *et al*. 2004, Coan *et al*. 2011, Chen *et al*. 2013, Sferruzzi-Perri *et al*. 2013, Kim *et al*. 2014).

We have demonstrated that a maternal LPD (9% casein) fed to mouse dams exclusively during preimplantation development (days 0–3.5 of gestation; Emb-LPD) enhances postnatal growth and adiposity and induces hypertension, vascular dysfunction and altered behavioural characteristics in adult offspring (Watkins *et al*. 2008, 2010, 2011). We observed additionally that enhanced early postnatal growth was a significant predictor of adult adiposity, cardiovascular health and behaviour (Watkins *et al*. 2008). Blastocysts transferred from LPD-fed dams into recipient NPD-fed dams displayed elevated fetal growth, when assessed at E17, demonstrating that offspring programming is initiated during preimplantation development (Watkins *et al*. 2008). Analysis of embryonic and extraembryonic lineage tissues from Emb-LPD-fed dams has revealed elevated blastocyst TE:ICM ratio and differential signalling through the mTORC1 nutrient sensing pathway (Eckert *et al*. 2012), enhanced endocytic and lysosomal activity within blastocyst TE and embryoid body primitive endoderm (Sun *et al*. 2014), and enhanced uptake and transport capacity within the late gestation visceral yolk sac (Watkins *et al*. 2008). From these data, we propose that the preimplantation embryo senses the maternal uterine nutritional status directly and initiates a series of adaptive mechanisms within the extraembryonic lineages to enhance nutrient uptake and maintain growth. However, upon restoration of optimal maternal nutrition post-implantation, these enhanced nutrient uptake adaptations become maladaptive, promoting increased fetal growth and adult offspring ill-health.

While the impact of maternal diet on embryonic and late gestation placental nutrition-uptake mechanisms has been investigated in detail, the nutritional impacts on early post-implantation dynamics, and how these processes influence the development of the mature placenta, remain unknown. Therefore, in the current study, we have extended our mouse gestational dietary model to investigate for the first time the impact of Emb-LPD on early post-implantation TGC phenotype *in vitro*, late gestation fetal growth and placental development.

## Materials and methods

### Animal treatments

All mice and experimental procedures were conducted using protocols approved by, and in accordance with, the UK Home Office Animal (Scientific Procedures) Act 1986 and local ethics committee at the University of Southampton. Virgin female MF-1 mice (aged 7–8.5 weeks), derived within the University's Biomedical Research Facility, were maintained on a 0700 h light:1900 h darkness cycle at a temperature of 20–22 °C and fed on standard laboratory chow *ad libitum* (Special Diet Services Ltd, Witham, Essex, UK), and were housed singly overnight with MF-1 studs. The presence of a vaginal plug the following morning was taken as a sign of mating. Plug-positive females were housed singly and allocated randomly to one of the three isocaloric (calories/g; Special Dietary Services Ltd; composition published previously (Watkins *et al*. 2011)) dietary regimens; i) normal protein diet (NPD; 18% casein, 42.5% maize starch, 21.3% sucrose, 10% corn oil, 5% cellulose), ii) LPD (9% casein, 48.5% maize starch, 24.3% sucrose, 10% corn oil, 5% cellulose) or iii) LPD from detection of a vaginal plug until day 3.5 of gestation, then switched to NPD for the remaining days of gestation (Emb-LPD). Females were culled via cervical dislocation at either E8.5, for the isolation and culture of post-implantation EPCs, or at day 17 (E17) for analysis of fetal growth and placental and yolk sac mTORC1 signalling.

### Isolation of EPC explants

In the mouse, E8.5 represents a developmental stage during which trophoblast differentiation and early placental lineage induction are being established. Therefore, analysis of EPC outgrowth phenotype at this stage could provide significant insights into the establishment of the mature placenta and the regulation of fetal growth in response to maternal diet (El-Hashash & Kimber 2004, El-Hashash *et al*. 2005). At E8.5, NPD-, LPD- and Emb-LPD-fed dams were culled by cervical dislocation. Whole uteri were excised and placed within pre-warmed (37 °C) DMEM (Life Technologies) with 10% (v/v) heat-inactivated FCS (Sigma–Aldrich). Individual decidual capsules were dissected from both uterine horns before isolation of whole embryos from within the surrounding decidual tissue. EPCs were separated from the embryo at the junction with the extraembryonic ectoderm using sterile fine forceps.

### Culture and outgrowth of secondary TGCs

Previously, we have demonstrated increased TE cell number and *in vitro* outgrowth expansion in blastocysts collected from LPD-fed dams (Eckert *et al*. 2012). To determine whether similar increases in expansive phenotype were still present within EPC outgrowths at E8.5, we cultured explants on coverslips to assess their growth within a two-dimensional plane. This approach enabled us to determine migratory phenotype while maintaining the close architectural structure of the outgrowth and preserving cell–cell contacts. Sterile coverslips were coated with BD Matrigel basement membrane matrix (BD Biosciences, Oxford, UK) diluted to 6 mg/ml in RPMI 1640 medium (Life Technologies) and left at 4 °C overnight in four-well plates (Sigma–Aldrich). The following morning, the Matrigel-RPMI medium was replaced with 500 μl sterile filtered RPMI 1640 medium (Life Technologies) containing 2% KnockOut Serum Replacement (Life Technologies), penicillin–streptomycin–glutamine mix (Life Technologies) and 26.2 μmol two-mercaptoethanol (Sigma–Aldrich) and allowed to equilibrate at 37 °C in an atmosphere of 5% CO_2_ for at least 1 h. Dissected EPC explants were cultured individually at 37 °C in an atmosphere of 5% CO_2_ for up to 48 h with a 50% medium change after 24 h.

### Assessment of EPC explant invasive capacity

In addition to cell migration, cell invasion is an important event in the development of the placenta. To obtain a greater understanding of the invasive capacity of outgrowths, we cultured EPC explants on Matrigel transwell inserts. Using this approach, we could quantitate invasive and migratory capacity through a three-dimensional membrane, replicating more closely an *in vivo* environment when compared with coverslips. Matrigel invasion assays of EPC explants were performed using BD BioCoat Matrigel Invasion Chambers (VWR, Lutterworth, UK). Transwell inserts (8 μm pore size) were re-hydrated within a 24-well plate with RPMI 1640 medium (Life Technologies) (500 μl/well and per insert) at 37 °C in an atmosphere of 5% CO_2_ for 2 h after which the medium was replaced with RPMI medium containing serum replacement, penicillin–streptomycin–glutamine and two-mercaptoethanol (as mentioned earlier in this study; 750 μl within the well and 500 μl within the invasion chamber). Invasion chambers were allowed to equilibrate at 37 °C in an atmosphere of 5% CO_2_ for at least 1 h. EPC explants, isolated as described earlier in this study, were cultured singly per well for up to 48 h with a 50% medium change after 24 h.

### Morphological and invasive analysis of outgrowths

EPC outgrowths, both on coverslips and invasion chambers, were fixed after 24 or 48 h of culture in 4% neutral buffered formalin (Sigma–Aldrich) for 15 min before permeabilisation with 0.1% Triton-X100 (Sigma–Aldrich) for 5 min, and washed three times with PBS at room temperature. Background staining was minimised with ammonium chloride (2.6 mg/ml in PBS) for 10 min before an additional 5 min permeabilisation step (0.1% Triton-X100) and PBS washing, all conducted at room temperature. Non-specific binding was blocked with 2% BSA in PBS containing 0.1% Triton-X100 for 30 min at room temperature before overnight incubation at 4 °C with a primary antibody for α-tubulin (1:2000; Cell Signaling, Danvers, MA, USA) in PBS with 1% BSA and 0.1% Triton-X100. Following washing in PBS with 0.1% Triton-X100, outgrowths were incubated with the appropriate Alexa Fluor conjugated secondary antibody (1:10 000; Molecular Probes, Life Technologies, Paisley, UK) for 1 h at room temperature and counterstained for nuclei using DAPI (Sigma–Aldrich) for 10 min before mounting on slides in DPX (Fisher, Loughborough, UK). All outgrowths were imaged using a Leica DSM 5000 microscope with epifluorescence capacity and analysed using the Volocity Software. The central EPC was defined as the central, single mass within which individual cell nuclei could not be determined. Proliferative trophoblast nuclei were defined as those located within the proximity of the central EPC and having a nuclear area <300 μm^2^ (Scott *et al*. 2000, El-Hashash & Kimber 2004). Secondary trophoblasts were defined as those located at the periphery of the outgrowth and possessing a nuclear area >300 μm^2^ (El-Hashash & Kimber 2004). Each outgrowth was assessed for the area of the EPC, proliferative and secondary trophoblast cells and of the whole outgrowth; distance of the furthest nucleus from the centre of the EPC; and perimeter of the outgrowth and number of secondary trophoblast cells.

### Analysis of fetal growth

At E17, NPD-, LPD- and Emb-LPD-fed dams were culled for the analysis of fetal growth. Whole uteri were removed, placed in ice-cold PBS and the number of conceptuses per horn recorded. Individual conseptuses were dissected carefully from the uterine tissue and weighed before separation of the fetus from the placenta and yolk sac. Each fetus, placenta and yolk sac were weighed individually before decapitation of the fetus for the collection of serum. Isolated placentae and yolk sacs were snap frozen in liquid nitrogen ahead of protein level analysis.

### Placental and yolk sac mTOR analysis

Individual placentae and yolk sacs were homogenised in lysis buffer (50 mM HEPES pH 7.4, 150 mM NaCl, 1 mM EDTA, 1 mM EGTA, 1% NP40; Sigma–Aldrich) containing 2% v/v protease inhibitor (complete mini protease inhibitor cocktail, Roche) and 1% v/v phosphatase inhibitor cocktails 1 and 2 (Sigma–Aldrich). Protein levels were determined using the DC protein assay kit (Bio-Rad) before boiling in LDS sample buffer (Life Technologies) and running on 4–12% bis–tris gels (Life Technologies). Proteins were transferred onto nitrocellulose membranes overnight, blocked in 5% milk in TBS-Tween (0.1% Tween 20; Sigma–Aldrich) before incubation overnight at 4 °C with primary antibodies (Cell Signaling) for total 4E-BP1 (1:1000), phospho- (Thr37/46) 4E-BP1 (1:1000), total S6 ribosomal protein (1:2000), phospho- (Ser235/236) S6 ribosomal protein (1:2000) and α-tubulin (1:10 000), all diluted in 5% BSA in TBS-Tween. After overnight incubation, membranes were washed in TBS-Tween before 1 h of incubation with appropriate species-specific, IRDye-conjugated secondary antibodies (Rockland, Limerick, PA, USA) in 5% milk in TBS-Tween20 at room temperature. Following additional washes with TBS-Tween20, membranes were analysed by densitometry using an Odyssey Infrared Imaging System (Licor, Lincoln, NE, USA). Proteins of interest were normalised to α-tubulin to control for differences in total protein loaded.

### Statistical analysis

All data were assessed for normality using the Shapiro–Wilk normality test (SPSS version 21). All fetal offspring organ weight data were analysed using a multilevel random effects regression model (SPSS version 21) to account for maternal origin of litter, gestational litter size, offspring sex and body weight where appropriate (Watkins *et al*. 2008). Analysis of placental and yolk sac protein levels and all EPC outgrowth measurements was performed by univariate ANOVA with Tukey's multiple comparison *post hoc* test for normally distributed data, or a Kruskal–Wallis test with Dunn's multiple comparisons *post hoc* test for non-normally distributed data (SPSS version 21). All data were assessed for interactions among maternal diet, offspring sex and litter size, where possible. Significance was considered at *P*<0.05.

## Results

### Characterisation of EPC explant outgrowth on Matrigel-coated coverslips

**Figure 1:**
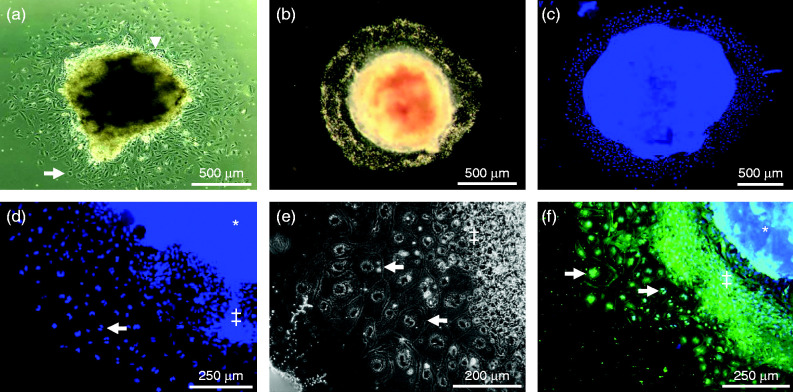
Representative images of E8.5 mouse EPC explant outgrowths grown on Matrigel-coated coverslips. (a) Inverted bright-field image taken under a Leica DSM 5000 microscope after 24 h showing small, densely packed cells and with elongated nuclei surrounding the central EPC (arrow head) and larger more widely spaced cells with rounded nuclei (arrow) at the periphery (shown at a higher magnification in d, e and f), or (b) top-illuminated bright-field image taken under a Zeiss Stemi SV11 stereomicroscope after 48 h in culture. (c) Whole image of DAPI-stained outgrowth after 48 h in culture. (d) Magnified section of an outgrowth showing the central EPC (*), small, closely associated ‘proliferative’ (‡) and peripheral ‘secondary’ (arrow) trophoblast giant cells in outgrowths after 48 h in culture. (e) Phase-contrast image of peripheral trophoblast giant cells (arrow) identifying a monolayer of large, multinucleated cells after 48 h of culture at the periphery of the ‘proliferative’ (‡) cells. (f) Immunofluorescence image stained for α-tubulin, showing multinucleated peripheral trophoblast giant cells (arrow) after 48 h of culture with proliferative layer (‡) and central EPC (*) shown on right.

At the time of cull (E8.5), the mean number of implantation sites per female (10.53±0.39) and the mean cross-sectional area of EPCs isolated for culture either on coverslips or in transwell inserts (0.83±0.04 mm^2^) did not differ between treatment groups (*P*>0.2). We first assessed the outgrowth characteristics of isolated EPC explants on coverslips coated with the basement membrane matrix Matrigel. After 24 h in culture, explants were observed to form a diffuse outgrowth, radiating in all directions from the central EPC. Visual inspection of outgrowths revealed them to be comprising two populations of cells; those immediately surrounding the central EPC, which appeared small, densely packed and with elongated nuclei, and those at the periphery, which were larger and possessed more rounded nuclei (Fig. 1a). After 48 h, outgrowths had increased in size but still comprised a central EPC surrounded immediately by small densely packed cells with larger cells populating the periphery (Fig. 1, b, c and d). Closer examination of these cell populations revealed the outer cells to be large (>1000 μm^2^), flat and multi-nucleated, while those cells surrounding the central EPC appeared smaller, multi-layered and possessed a single nucleus (Fig. 1e and f). Previous studies have identified the cells closest to the central EPC explant as being proliferative stem cells (Scott *et al*. 2000, El-Hashash & Kimber 2004), while those cells located at the outgrowth periphery display morphological characteristics of TGCs (large, multinucleated and single layered) and express specific markers such as PL-II, not expressed within proliferating cells (El-Hashash & Kimber 2004).

**Figure 2:**
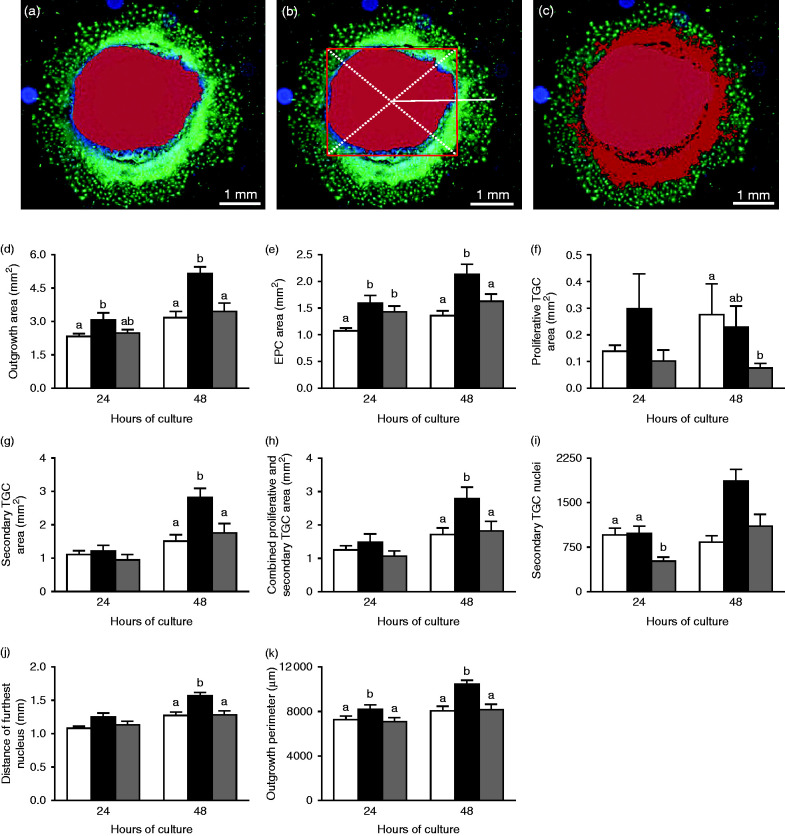
(a, b and c) Example of E8.5 mouse EPC explant outgrowth analysis using the Volocity Software to determine EPC area (highlighted in red in a) and distance of the furthest nucleus from the EPC centre (solid white line from the centre of the superimposed square in b) and the combined area of the EPC and proliferative cells (highlighted in red) in (c). The area of the secondary TGCs was determined as the areas of the entire outgrowth minus the combined area of the EPC and proliferative TGCs. (d, e, f, g, h, i, j and k) Mean EPC outgrowth measurements from NPD (white bars), LPD (black bars) and Emb-LPD (grey bars) explants grown on Matrigel-coated coverslips after 24 and 48 h. *n*=10–14 outgrowths taken from 6–8 separate females per diet per culture time and treatment group. Error bars are s.e.m. Different letters denote statistical significance between groups at *P*<0.05.

Using these morphological characteristics, we defined mean fluorescence intensity (α-tubulin) and nucleus area (DAPI) threshold values to define the area of the central EPC (Fig. 2a). To determine the distance of the furthest nucleus from the centre of the EPC, a square boundary encompassing the entire highlighted EPC was superimposed and the centre of this square determined by two diagonal lines connecting each corner. From the crossing point of these two lines, the distance of the furthest nuclei could be determined (Fig. 2b). In addition, we determined the area of proliferative (Fig. 2c) and secondary TGCs as well as the total area and perimeter of each outgrowth. These standardised threshold values were then applied to each outgrowth image to provide an objective set of measurement criteria. Analysis of EPC explants after 24 h revealed that LPD outgrowths had a significantly larger mean total area (i.e. comprising central EPC and surrounding proliferative and secondary TGCs) than NPD outgrowths (*P*=0.05) and a trend to exceed that of Emb-LPD outgrowths (*P*=0.092) (Fig. 2d). Both LPD and Emb-LPD outgrowths had significantly increased mean EPC area after 24 h when compared with NPD outgrowths (Fig. 2e) (*P*<0.01). No differences in mean area of proliferative or secondary TGCs, or in their combined area, were observed between groups after 24 h (Fig. 2f, g and h). However, Emb-LPD outgrowths had significantly reduced mean number of secondary TGC nuclei when compared with NPD and LPD outgrowths (Fig. 2i) (*P*<0.01). Despite no significant difference in the mean distance of the furthest cell from the centre of the EPC being observed between groups (Fig. 2j), LPD outgrowths displayed an increased outgrowth perimeter when compared with NPD and Emb-LPD outgrowths (Fig. 2k) (*P*<0.05).

After 48 h of culture on Matrigel-coated coverslips, LPD outgrowths displayed significantly elevated mean total outgrowth area (Fig. 2d), EPC area (Fig. 2e), area of secondary TGCs (Fig. 2g), combined proliferative and secondary TGC area (Fig. 2h), number of secondary TGC nuclei (Fig. 2i), distance of the furthest nucleus from the EPC centre (Fig. 2j) and outgrowth perimeter (Fig. 2k) when compared with outgrowths from both NPD- and Emb-LPD-fed females (*P*<0.05). Outgrowths from Emb-LPD-fed females displayed a significantly reduced mean area of proliferative cells when compared with outgrowths from NPD-fed females after 48 h (Fig. 2f) (*P*=0.006) and found to be at a trend level (*P*=0.074) when compared with LPD outgrowths.

**Figure 3:**
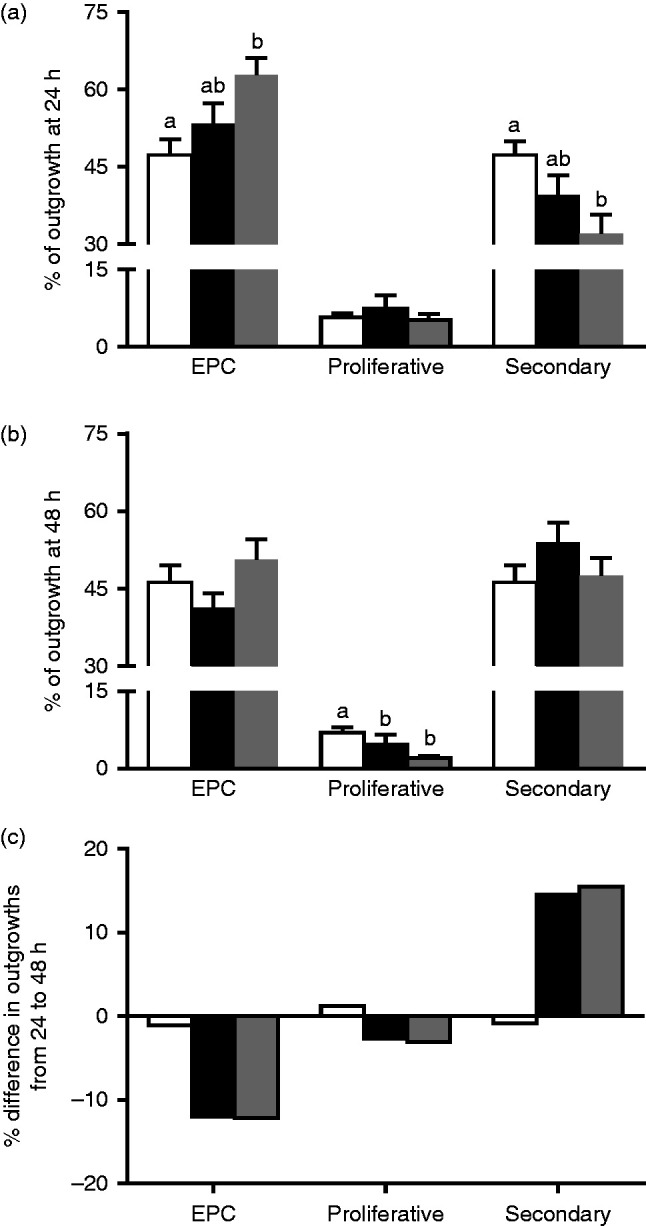
(a) Mean % composition of NPD (white bars), LPD (black bars) and Emb-LPD (grey bars) explants grown on Matrigel-coated coverslips after 24 and (b) 48 h. (c) Mean change in outgrowth composition between 24 and 48 h of culture. *n*=10–14 outgrowths per culture time and treatment group, taken from 6–8 separate females per diet. Error bars are s.e.m. Different letters denote statistical significance between groups at *P*<0.05.

Using data collected on outgrowth proportions for each dietary group between 24 and 48 h in culture, we assessed the relative development and changes in EPC, and proliferative and secondary TGC composition. After 24 h of culture on Matrigel-coated coverslips, outgrowths from Emb-LPD-fed females were composed of a significantly higher EPC and reduced secondary TGC proportion when compared with outgrowths from NPD-fed females (Fig. 3a) (*P*<0.05). After 48 h of culture, no significant differences in mean EPC or secondary TGC proportions were observed between groups (Fig. 3b). However, significantly reduced proliferative TGC proportions were observed for outgrowths from females fed LPD and Emb-LPD when compared with NPD-fed females (Fig. 3b) (*P*<0.05). This alteration in outgrowth composition between 24 and 48 h of culture resulted from significant reductions in mean EPC proportions (LPD 11.99%, Emb-LPD 12.20%) and increased secondary TGC proportions (LPD 14.50%, Emb-LPD 15.49%), while the NPD outgrowths altered their EPC and secondary TGC proportions by 1% or less (Fig. 3c).

### Characterisation of EPC explant invasive phenotype

**Figure 4:**
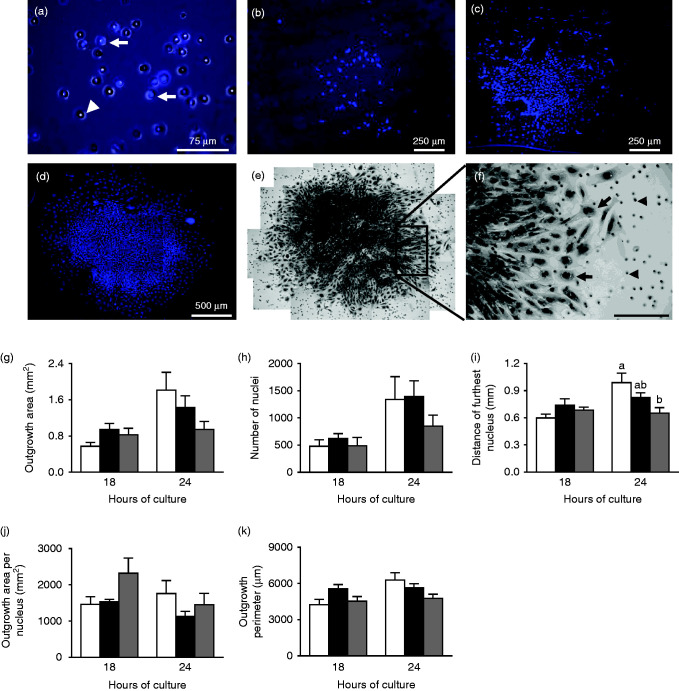
(a) High-magnification image of a DAPI-stained Transwell invasion chamber showing cells migrating (arrows) though the 8 μm membrane pores (arrow head) after 6 h in culture. (b) Lower magnification image of a DAPI-stained Transwell invasion chamber after 6, (c) 12 and (d) 18 h of culture. (e) Haematoxylin-stained outgrowth after 18 h of culture. (f) Magnified section showing large, multinucleated peripheral cells (arrows) and 8 μm pores (arrow heads). (g, h, i, j and k) Mean EPC outgrowth measurement from NPD (white bars), LPD (black bars) and Emb-LPD (grey bars) explants grown on Matrigel invasion transwell inserts after 18 and 24 h. *n*=6–7 outgrowths per culture time and treatment group, taken from 6–7 separate females per diet. Error bars are s.e.m. Different letters denote statistical significance between groups at *P*<0.05.

In addition to our analysis of EPC explant outgrowth on coverslips, we also assessed explant invasive capacity *in vitro* using Matrigel-coated transwell inserts. After 6 h, we observed that relatively few cells had penetrated the Matrigel coating, migrating through the 8 μm pores (Fig. 4a) and spreading over the under-surface of the insert (Fig. 4b). The number of cells observed to have migrated to the under-surface of the insert increased between 12 h (Fig. 4c) and 24 h (Fig. 4d) such that, following 24 h in culture, a large population of cells were present on the under-surface of the insert. Closer examination of these invasive and migratory cells after 24 h of culture revealed a central mass of densely packed, predominately mono-nucleated cells, with a single layer of larger, multinucleated cells at the periphery (Fig. 4e and f), reflective of outgrowths cultured on coverslips. After 36 h of culture, the large number of cells present on the under-surface of the insert prevented identification of individual nuclei and, therefore, quantitative determination of cell migration and outgrowth invasive characteristics (data not shown). Therefore, we analysed outgrowth invasive phenotype after 18 and 24 h of culture, measuring the total outgrowth area and perimeter, the number of nuclei and the distance of the furthest nuclei from the centre of the outgrowth.

After 18 h in culture, no significant difference in the mean outgrowth area, number of nuclei, distance of the furthest nucleus, outgrowth area per nucleus or perimeter was observed between treatment groups (Fig. 4g, h, i, j and k). After 24 h in culture, Emb-LPD outgrowths displayed a significantly reduced mean distance of the furthest nucleus from the outgrowth centre (Fig. 4i; *P*=0.017) and reduced mean outgrowth area and perimeter at trend levels (Fig. 4g and e; *P*<0.01).

### Fetal development at E17

Previously, we have demonstrated elevated fetal growth at E17 in offspring derived from blastocysts transferred from LPD-fed dams into NPD-fed dams (Watkins *et al*. 2008). This significantly increased growth reflected the weight at birth and throughout life of Emb-LPD-fed offspring. Therefore, to determine whether maternal diet influenced fetal development in the current study, we evaluated fetal growth at the same stage (E17) of gestation (Table 1). At the time of cull (E17), there was no significant difference in the mean litter size between treatment groups (NPD 12.86±0.95, LPD 13.31±0.37, Emb-LPD 11.15±0.50; *P*>0.05). Concepti from Emb-LPD-fed dams were significantly heavier than those from NPD- and LPD-fed dams (*P*<0.05). Following dissection, Emb-LPD-fed fetuses also displayed elevated weight when compared with NPD- and LPD-fed fetuses (*P*<0.05). Placentas from LPD-fed fetuses were significantly lighter than those of NPD- and Emb-LPD-fed fetuses (*P*<0.05). The significantly increased Emb-LPD fetal weight and decreased LPD placental weight resulted in significantly increased fetal:placental ratios in both groups when compared with NPD-fed offspring. Although no difference in the mean yolk sac weight has been observed between dietary groups, Emb-LPD-fed fetuses displayed a significantly elevated fetal:yolk sac ratio when compared with NPD- and LPD-fed fetuses (*P*<0.01).

### E17 placental and yolk sac mTOR protein analysis

**Figure 5:**
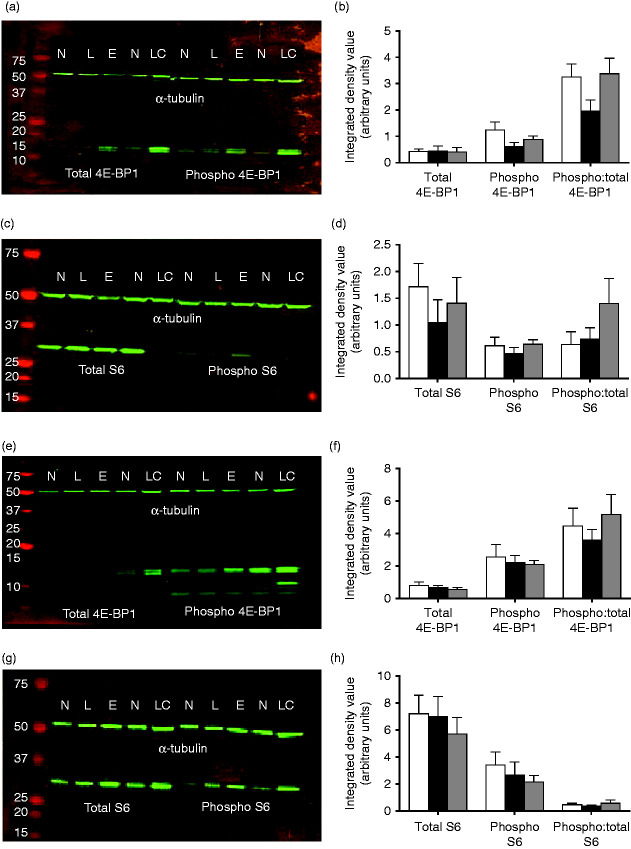
Representative immunoblots in E17 (a and c) placenta and (e and g) yolk sac tissues for mTORC1 downstream targets of total and phosphorylated (a and e) 4E-BP1 and (c and g) S6 protein levels and the reference protein α-tubulin. Individual blot lanes include molecular weight markers (far left) and samples of NPD (N), LPD (L) and Emb-LPD (E) tissues (20 μg total protein per lane) and loading control (LC). Mean integrated density values for total and phosphorylated levels of (b and f) 4E-BP1 and (d and h) S6 ribosomal protein in NPD (white bars), LPD (black bars) and Emb-LPD (grey bars) E17 (b and d) placental and (f and h) yolk sac tissues normalised to α-tubulin. *n*=8 samples per treatment, all from separate litters. Error values are s.e.m.

Changes in placental size, function and mTORC1 activity in response to maternal gestational diet have been demonstrated previously (Rosario *et al*. 2011, Jansson *et al*. 2012). Therefore, we assessed the total and phosphorylated levels of the downstream targets of mTORC1, 4E-BP1 and S6 ribosomal protein, in placental and yolk sac tissues. 4E-BP1 acts as a translation repressor protein by binding to the translation initiation factor eIF4E, an interaction inhibited by the phosphorylation of 4E-BP1 (Dowling *et al*. 2010). Conversely, the phosphorylation of S6 ribosomal protein, mediated by phosphorylated S6 kinase, results in the translation of terminal oligopyrimidine-dependent transcripts, encoding ribosomal proteins involved in cell cycle progression and translation regulation (Kim 2009). Analysis of total and phosphorylated 4E-BP1 and S6 ribosomal protein levels in offspring placental and yolk sac tissue revealed no significant differences between treatment groups (Fig. 5). However, a trend towards a reduced level of phosphorylated 4E-BP1 and a decrease in the ratio of phosphorylated to total 4E-BP1 in LPD placentas was observed when compared with NPD placentas (*P*=0.077 and 0.099 respectively). Similarly, decreased levels of total and phosphorylated S6 ribosomal protein were observed in LPD placentas when compared with NPD and Emb-LPD placentas, though these did not reach significance. No differences were observed in the levels of 4E-BP1 or S6 ribosomal protein in offspring yolk sac tissue between dietary groups.

## Discussion

Appropriate placental development and function plays a pivotal role in directing fetal growth, offspring phenotype and influencing adult predisposition to cardiovascular and metabolic disease risk (Vaughan *et al*. 2011). During early mammalian development, TGCs are fundamental for the establishment of maternal–fetal nutrient and gas exchange and in the formation of the placenta (Rossant & Cross 2001). As the post-implantation EPC is considered the primary source of secondary TGCs, the *in vitro* culture of the EPC provides a system in which early placental dynamics can be assessed in response to maternal nutritional status. In this study, we investigated the impact of maternal low-protein dietary regimes on post-implantation trophoblast spreading phenotype and invasive capacity *in vitro*, fetal development and signalling through the nutritional sensing mTORC1 pathway in late gestation placenta and yolk sac tissues. We observed that maternal LPD enhanced the development and growth of EPC explants taken at E8.5, but that their invasive capacity remained unaltered when compared with explants taken from NPD- and Emb-LPD-fed dams. In late gestation (E17), both maternal LPD and Emb-LPD treatments induced significant increases in the fetal:placental ratio, through reduced placental and enhanced fetal growth respectively. Signalling responses mediated by the mTORC1 pathway within the placenta and yolk sac tissues were similar across all treatment groups. These results expand our understanding of the early developmental mechanisms regulating fetal growth and development in response to maternal nutrition, providing novel insights into the programming of adult offspring health.

Mouse TGC differentiation has been characterised in detail, revealing a complex expression pattern of blood group antigens (B and Le-b/Le-y), integrins (α_7_), hormones (PL-II, PTHrP) and transcription factors (AP-2γ) both *in vitr* *o* and *in vivo* (Centrella *et al*. 1989, Brown *et al*. 1993, Auman *et al*. 2002, El-Hashash *et al*. 2010). It has been demonstrated that cells with large nuclear area (>300 μm^2^) located at the periphery of EPC outgrowths express PL-II and AP-2γ, identifying these as secondary TGCs (El-Hashash & Kimber 2004). In contrast, cells located immediately adjacent to the EPC have been shown to be proliferative stem cells (Scott *et al*. 2000). Therefore, using these pre-defined morphological (cell size, nuclear area, single or multi-layered) and locational (peripheral or adjacent to the EPC) characteristics, we quantitatively measured EPC explant outgrowth *in vitro*. EPC explants from all treatments groups outgrew in a radial fashion, retaining a central EPC mass surrounded by small, dense, round proliferative cells. At the periphery of each outgrowth were large, flat, multinucleated cells. Upon quantification of each of these outgrowth regions, we observed that EPC explants cultured from LPD-fed dams displayed significantly larger EPC and secondary TGC areas with increased numbers of secondary TGC nuclei when compared with NPD and Emb-LPD outgrowths after 24 and 48 h. The increase in secondary TGC area appeared to be driven by increased rates of cell division, as indicated by an increased number of nuclei. However, a disproportionate increase in the number of large multi-nucleated secondary TGCs in LPD outgrowths cannot be discounted. In addition, the contribution of cells from the proliferative region was not assessed, and so an increase in cell allocation from this area cannot be dismissed. However, as no significant differences in mean proliferative TGC area were observed, the contribution to the increase in secondary TGC area may be small. Interestingly, we observed modest increases in EPC outgrowth in both NPD and Emb-LPD explants, differing from each other in EPC area (increased in Emb-LPD) and number of secondary TGCs (decreased in Emb-LPD) at 24 h only. Therefore, the up-regulation of EPC outgrowth phenotype in response to maternal LPD could provide a more metabolically efficient early gestation adaptive mechanism to promote enhanced embryonic-maternal vascular connection and maintain fetal development rather than increasing placental mass in late gestation. Indeed, changes in extraembryonic lineage allocation and spreading phenotype have been identified in blastocysts collected from LPD-fed mouse and rat dams (Kwong *et al*. 2000, Eckert *et al*. 2012). In addition, enhanced endocytotic and lysosomal activity within LPD blastocyst TE and embryoid body primitive endoderm has also been reported (Sun *et al*. 2014). Similar adaptive responses have been shown to occur in response to maternal diabetes, where enhanced rates of cell proliferation have been reported in EPCs and late gestation placentas collected from diabetic rat dams, while the culture of control dam EPCs in high glucose media increased rates of EPC spreading (Caluwaerts *et al*. 2000, Zorn *et al*. 2011).

To determine whether maternal diet affected outgrowth invasive capacity, we cultured isolated EPCs on Matrigel-coated porous membranes, allowing invasive cells to penetrate the gel and migrate to the lower surface. Matrigel assays have been used to characterise the invasive phenotype of human trophoblast cells (Sato *et al*. 2002, Xu *et al*. 2002, O'Brien *et al*. 2003), revealing differential invasive responses to a range of pro-inflammatory cytokines (Staff *et al*. 2000, Bauer *et al*. 2004, Pavan *et al*. 2004, Jovanovic *et al*. 2010). In mouse assays, TGCs have been shown to migrate through the insert ahead of smaller cell types (Hemberger *et al*. 2004). *In vivo*, altered patterns of trophoblast invasion have been reported in a rat model of maternal gestational obesity (Hayes *et al*. 2014). We observed that after 6–12 h in culture, relatively few cells had migrated to the lower surface of the membrane; however, after 36 h in culture, it was not possible to identify individual nuclei. Therefore, in order to characterise invasive and migratory phenotype, we assessed outgrowths after 18 and 24 h in culture. In contrast to our findings of EPC outgrowth phenotype on Matrigel-coated coverslips, we observed no difference in EPC explant invasive characteristics, with respect to maternal diet, when assessed using transwell inserts. This difference could be accounted for by the fact that, on coverslips, outgrowing cells can only migrate away radially and in a single plane from the central EPC; however, with transwell inserts, cells can disperse to the lower surface and migrate in any direction resulting in more diffuse outgrowth patterns. As it was only possible to analyse the lower surface of the transwell insert, it was not possible to assess the proportions of cells remaining on the upper surface, or which may still be migrating through the membrane. As such, for future studies increasing the number of analytical time points, assessing for specific markers of secondary TGCs, determination of gene and protein expression patterns within our outgrowths and/or assessing TGC development *in vivo* would all provide additional insights into the invasive characteristics of EPC outgrowths in response to maternal diet. In addition, as our outgrowths were cultured under ambient oxygen condition, as apposed to the more physiological low oxygen tension observed *in utero*, we cannot rule out potential confounding effects on TGC phenotype in response to this disparity. As such, future *in vitro* studies will employ conditions more physiologically relevant to mimic better those observed *in utero*.

It would be anticipated that any adaptive mechanisms initiated within LPD-fed pre-implantation embryos would also be induced within Emb-LPD-fed embryos. It was of interest to observe significant differences in outgrowth phenotype between LPD and Emb-LPD EPCs cultured on coverslips. Analysis of changes in outgrowth proportions over time did, however, reveal that LPD and Emb-LPD explants behaved in a similar manner, reducing the relative proportion of the EPC and increasing the relative proportion of secondary TGCs. This was in stark contrast to NPD outgrowths, which remained proportionally unchanged over the same period. As discussed earlier, we proposed that maternal LPD induces nutrient retrieval mechanisms within the extraembryonic lineages of the developing embryo. Therefore, maternal LPD may induce the blastocyst to invest disproportionately into the developing EPC after implantation. If the maternal diet remains sub-optimal, then the adaptive investment increases rates of cell division and nutrient uptake in order to maintain fetal development under conditions of reduced nutrient availability. However, when maternal NPD is restored at implantation (Emb-LPD), rates of trophoblast cell division are unaltered, but the programmed enhanced nutrient uptake adaptations result in increased fetal growth.

The development and size of the late gestation placenta is directly linked to nutrient transport capacity and fetal growth. Placental morphology, thickness, fetal–placental blood flow and the expression and function of nutrient transporters also influence fetal growth and development (Salafia *et al*. 2007). In addition to the placenta, the visceral yolk sac provides the developing fetus with a constant supply of amino acids through the breakdown of maternal proteins throughout gestation. Therefore, we determined whether changes in post-implantation trophoblast phenotype affected fetal, placental and yolk sac development in late gestation (E17). Our analysis revealed elevated fetal growth in offspring from Emb-LPD-fed dams, reflecting our previous findings of elevated birth weight of Emb-LPD-fed offspring and of late gestation fetuses derived from LPD blastocysts transferred into NPD-fed dams (Watkins *et al*. 2008). In addition, we observed a significantly elevated fetal:yolk sac weight ratio in Emb-LPD-fed fetuses when compared with NPD- and LPD-fed fetuses. Maternal LPD in the mouse has been shown to enhance nutrient uptake activity within the blastocyst TE and embryoid body primitive endoderm (Sun *et al*. 2014) and elevate visceral yolk sac endocytosis in late gestation (Watkins *et al*. 2008). Therefore, the enhanced fetal growth observed in Emb-LPD-fed offspring could be programmed in part through increased delivery of essential nutrition by the yolk sac during gestation. Conversely, fetuses from LPD-fed dams displayed comparable fetal weight but with a significantly reduced placental weight. The significantly increased Emb-LPD fetal weight, and decreased LPD placental weight resulted in significantly elevated fetal:placental weight ratios in both LPD- and Emb-LPD-fed fetuses. The fetal:placental ratio is conceived as an indicator of placental nutrient transport capacity, with an increased ratio considered as a sign of enhanced placental efficiency (Fowden *et al*. 2009). As discussed earlier in this study, in response to maintained maternal LPD, it may be more metabolically favourable to establish placentation earlier and minimise placental growth in later gestation when maternal resources are constrained. However, this adaptation may then result in altered placental function and transport, having important consequences for offspring growth and adult health. Maternal LPD in rats has been shown to result in the down-regulation of placental amino acid transporters ahead of development of intrauterine fetal growth restriction (Malandro *et al*. 1996, Jansson *et al*. 2006). Sferruzzi-Perri *et al*. (2013) demonstrated that a maternal obesogenic diet reduced fetal and placental growth, but enhanced placental glucose and amino acid transport, as well as expression of fatty acid transporters and metabolic signalling pathway mediators. The molecular regulation of placental nutrient transport is believed to be coordinated through mTORC1, acting as a central nutrient sensor orchestrating fetal demand with maternal supply (Jansson *et al*. 2012). Maternal gestational nutrient restriction has been shown to decrease placental mTORC1 activity in the baboon (Kavitha *et al*. 2014) and human IUGR pregnancies (Roos *et al*. 2007, Yung *et al*. 2008), and in response to rodent LPD (Jansson *et al*. 2006, Rosario *et al*. 2011). In addition, circulating levels of insulin and branched chain amino acids, activators of mTORC1, are altered in models of maternal dietary induced obesity (Gaccioli *et al*. 2013, Lager *et al*. 2014). Therefore, we investigated the expression of key downstream targets of mTORC1 and their phosphorylation status in placentas from LPD- and Emb-LPD-fed dams. Herein, we observed minimal impacts of maternal diet on signalling through the mTORC1 pathway as assessed by the total and phosphorylated levels of the initiation factor eIF4E and S6 ribosomal protein in either placental or yolk sac tissues. While significant changes in maternal metabolite and growth factor levels have been shown at the time of implantation in response to maternal LPD (Kwong *et al*. 2000, Eckert *et al*. 2012), the demonstration of late gestation maternal autophagy in rat dams fed LPD (Wang *et al*. 2014) raises the possibility that maternal nutritional status stabilises after several days of LPD, resulting in the similar levels of mTOC1 signalling observed in this study. In addition, as physiological exchange between maternal and fetal circulations occurs within the labyrinthine zone of the placenta, our analysis of mTOR signalling within the whole placenta may have masked zone-specific differences. Finally, the placenta may augment other mechanisms independently of mTOR to modify nutrient transport, thus resulting in similar levels of mTOR signalling, but still affecting fetal development. For example, in nutrient-restricted sheep at mid-gestation, a reduction in fetal growth is observed with increased AMPK and ERK1/2 activity but with no change in mTOR and Akt signalling (Ma *et al*. 2011).

Taken together, our results indicate that maternal LPD programmes post-implantation trophoblast growth and phenotype in order to maintain embryonic and fetal development within a sub-optimal nutritional environment. In late gestation, the maintenance of maternal LPD results in a significantly smaller placenta but with comparable fetal weight, suggestive of enhanced placental efficiency and transport. However, these adaptive mechanisms, while supporting offspring development to birth and reproductive age, become maladaptive and predispose offspring to adult-onset disease (Watkins *et al*. 2008). In contrast, maternal Emb-LPD does not affect early post-implantation trophoblast spreading and cell number, due potentially to the restoration of maternal nutrition to optimal (NPD) levels after implantation. However, the induction of enhanced nutrient uptake capacity, programmed within the blastocyst in response to preimplantation LPD, results in elevated fetal growth in late gestation, also inducing adult cardiovascular and metabolic disease (Watkins *et al*. 2008). These data add new insights into the sensitivity of the developing embryo in response to maternal nutrition at the time of embryo implantation, for the programming of offspring growth and subsequent health.

## Figures and Tables

**Table 1:** Mean weights of concepti and fetal tissues from NPD-, LPD- and Emb-LPD-fed mouse dams at day 17 of gestation.

**Tissue**	**Diet**		
NPD	LPD	Emb-LPD
Conceptus (mg)	1261.7±15.9^a^	1227.6±18.5^a^	1324.2±18.2^b^
Fetus (mg)	941.2±8.6^a^	943.4±9.4^a^	1039.5±9.3^b^
Placenta (mg)	169.2±2.5^a^	150.1±1.9^b^	159.7±2.2^a^
Yolk sac (mg)	82.2±1.6	87.6±1.6	83.03±2.0
Fetal:Placental	5.74±0.09^a^	6.40±0.09^b^	6.64±0.09^b^
Fetal:Yolk sac	12.37±0.32^a^	11.46±0.29^a^	13.61±0.38^b^

*n*=13–14 dams per treatment group. Error values are s.e.m. Different letters denote statistical significance at *P*<0.05.
